# A Case of Cerebro Spinal Hyperæmia

**Published:** 1892-09

**Authors:** A. C. Moreland

**Affiliations:** Atlanta, Georgia


### A CASE OF CEREBRO-SPINAL HYPERÆMIA.

BY A. C. MORELAND, M. D.,
ATLANTA, GEORGIA.

In March of the present year I was summoned to a sudden attack of illness in a young man about nineteen years of age. Four days prior to the first visit, the patient had taken a full dose of calomel with decided results. Diet was never considered.

For two days prior to the attack dizziness occurred, the appetite waned, and alternate flushing and paleness was present. Relative to the quality and quantity of food consumed, he ate largely and usually of wheaten breads, syrup and tea. Gravies were (fortunately) excluded. Rapid eating was his custom. In view of the influences that habits have upon disease, it is best to state that the patient was a free consumer of cigarettes. Constipation had become second nature. Examination revealed a pulse of 120, temperature of 99, lancinating pains in abdomen, chest, head and spine, followed by clonic paroxysms every three minutes, with tendency to opisthotonos.

Chilly sensations darted at random over the body, the hands and feet were cold, the head and along the spine was uniformly hot. Respiration was first very fast, soon becoming slow. The epidermis of scalp, face and along spine was very red and intensely hyperæsthetic. The pupils were normal. The sclera was congested. Photophobia was not present. Tinnitus aurium was pronounced, and the least noise was most painful. The tongue was enlarged and coated white. The abdomen was distended and very tympanitic. The physical condition was plethoric with decided tendency to obesity.

In studying this case, we must find the causes of the constipation. If this can be fully established, we then will have the key to all the symptoms and can easily diagnosticate the case. As all constipation is the result of an imperfect digestion, we can reasonably hope to find the solution to our proposition in the item of diet with the environments of habit.

The history of the case shows that our patient used excessively and exclusively wheaten breads, syrup (and other saccharine foods) and tea. Can there be a diet better adapted to the production of fermentative dyspepsia ? We are told that tea is composed of theine, aromatic oil, sugar, gum and tannic acid ; then we have (in various forms) a saccharine diet to accompany this dyspeptic beverage.

The results of such a continued alimentation are found in the present case. The bowels are tympanitic, the face is flushed deeply, slight noise produces great discomfort, clonic seizures are frequent, and reflex pains play at random over the body. Supper had just been indulged. And now, though the fever was slight, we find the stomach full of a belabored supper, diligently in search of peptones. The smaller intestines are yet engaged with an unwieldy dinner. Fermentation takes place in the alimentary tract, and a pressure upon the abdominal aorta and branches by distension results. A cerebro-spinal hyperæmia ensues.

Having reviewed the history, symptoms and physical examination of the case, we must now apply our therapy to remove the cause. The first indication which claimed my attention was the removal of the undigested contents of the stomach and intestines. This was done by the free use of ipecac, calomel and warm enemas. Enormous quantities of scybalæ were passed. The patient did not then enjoy a refreshing doze, but the reflexes were not so aggravated. Morphia (gr. $\frac{1}{8}$) and atrophia (gr. $\frac{1}{200}$) was next given to allay the erratic pains resulting from the acute indigestion present. The effect was the relief of the opisthotonotic attacks and the reduction of the clonic paroxysms to every seven minutes.

The heart beat was still rapid, and the slight febrile condition remained unchanged. We then had recourse to aconitine and strophanthus, the one to control the acute febrile action, the other to regulate the cardiac stroke. These results were had promptly. Synchronously with the administration of aconite and strophanthus, a sinapism (full strength) was applied to the entire length of spine. Not until the aconite and cataplasm was used did the intense hyperæsthesia abate, and with the lowering of temperature, regulation of pulse and cessation of hyperæsthesia, the clonic paroxysms were controlled. Only a general soreness remained, which finally yielded to hot turpentine cloths.

With cessation of the various reflexes, the bladder discharged a large amount of limpid urine. The respiration became normal, and the dormant desire for cigarette smoking again asserted itself.

So far we have dealt with the direct issue of the case, but there are tangents to which I wish to call your attention. First, the toxic effects of tea; second, the toxic effects of tobacco; third, the atmospheric influence on the case.

The history of the case reveals flatulent constipation, vertigo and tinnitus aurium. These I have credited to the excessive use of tea, but not tea alone, for we have the tobacco habit to reason upon. Tobacco excessively used will also produce giddiness, and I judge that the clonic convulsions were directly due to tobacco.

We have seen how the excessive use of wheaten bread, syrup, tea and tobacco have affected the case. But there is another element in the case not before mentioned—the atmospheric condition.

During this time, if my readers will refer to their case book, they will find that la grippe was abroad in the land. Every "cold" was possessed of some peculiarity. In the present instance I am convinced that the acute indigestion and the slightly elevated temperature and the opisthotonotic tendency was due to an atmospheric impress made upon the system while laboring under chronic constipation, which is already causing a fluxionary hyperæmia.

On the third day after attack the patient dressed, though remaining close in his room.

A free dose of Epsom salts was given to flush the primæ viæ, after which a liquid diet was ordered and the following given as an after treatment :

℞. Potass. iodidi....................................ʒiii.

Ex. cascaræ sag. fl.,

Ex. rhei. fl.,.................aa................℥i.

Aq. menthæ pip...............................℥ii.

Infus. digitalis...................................℥iv.

M. Sig.—Shake. Take a teaspoonful after meals.

